# Layer Contour Verification in Additive Manufacturing by Means of Commercial Flatbed Scanners

**DOI:** 10.3390/s20010001

**Published:** 2019-12-18

**Authors:** David Blanco, Pedro Fernandez, Alvaro Noriega, Braulio J. Alvarez, Gonzalo Valiño

**Affiliations:** Department of Construction and Manufacturing Engineering, University of Oviedo, c/Pedro Puig Adam, E.D.O.5, 33203 Gijon, Asturias, Spain; pedrofa@uniovi.es (P.F.); noriegaalvaro@uniovi.es (A.N.); braulio@uniovi.es (B.J.A.); gvr@uniovi.es (G.V.)

**Keywords:** additive manufacturing, flatbed scanner, layer contour verification

## Abstract

Industrial adoption of additive manufacturing (AM) processes demands improvement in the geometrical accuracy of manufactured parts. One key achievement would be to ensure that manufactured layer contours match the correspondent theoretical profiles, which would require integration of on-machine measurement devices capable of digitizing individual layers. Flatbed scanners should be considered as serious candidates, since they can achieve high scanning speeds at low prices. Nevertheless, image deformation phenomena reduce their suitability as two-dimensional verification devices. In this work, the possibilities of using flatbed scanners for AM contour verification are investigated. Image distortion errors are characterized and discussed and special attention is paid to the *plication* effect caused by contact imaging sensor (CIS) scanners. To compensate this phenomena, a new local distortion adjustment (LDA) method is proposed and its distortion correction capabilities are evaluated upon actual layer contours manufactured on a fused filament fabrication (FFF) machine. This proposed method is also compared to conventional global distortion adjustment (GDA). Results reveal quasi-systematic deformations of the images which could be minimized by means of distortion correction. Nevertheless, the irregular nature of such a distortion and the superposition of different errors penalize the use of GDA, to the point that it should not be used with CIS scanners. Conclusions indicate that LDA-based correction would enable the use of flatbed scanners in AM for on-machine verification tasks.

## 1. Introduction

Additive manufacturing (AM) encompasses a wide range of processes whose common characteristic is that three-dimensional parts are built from two-dimensional layers deposited on top of each another. During the last decades, AM has experienced a continuous development, evolving from merely part prototyping to full-functional part manufacturing. Nevertheless, although industrial development of AM is not expected to reach the plateau of productivity until the next decade [1], current figures indicate that industrial AM systems have experienced a great impulse during last few years, whereas sales of desktop 3D printing systems (under $500) are clearly declining [2].

Generalized industrial adoption of AM has to face several challenges, like broadening the range of available materials, increasing production batch size or improving part quality [3]. Although part quality is a broad concept that encompasses aspects such as physical properties or durability, dimensional and geometrical accuracy of AM parts have always stood out among researchers’ main concerns during the last decade [4,5,6,7,8]. These works analyzed the lack of dimensional or geometric quality in three-dimensional features in final parts, which enabled optimization of process configuration, or even for the modification of part design, so that an improvement in quality could be achieved in the following parts. Although this is the usual approach for quality assurance in AM it does not permit adoption of close-loop in-process improvements, and implies that optimization could not be done without previously manufacturing test specimens.

Nevertheless, regarding the dimensional quality of manufactured parts, one key achievement would be to build layer contours that accurately follow theoretical profiles. This possibility would require on-machine integration of measurement devices to evaluate in-layer quality. The use of sensors capable of verifying each individual layer and checking the accuracy in contour tracing would allow for correction of the next layers. They would also make unnecessary previous testing of each particular geometry. Therefore, real-time, in-line metrology is commonly reported to be among the main challenges for AM development [9]. Contour verification could be carried out by means of different technologies, like structured light [7], conoscopic holography [10], coordinate measuring machine (CMM) optical probes [11] or ad hoc Charge-Couple Device (CCD) based instrumentation [12,13]. Nevertheless, computer vision based on flatbed scanner images should be considered as a serious candidate, since it can meet high scanning speeds at low prices.

Flatbed scanners are optical devices commonly used to capture digital images of flat elements, such as sheets of paper or photographs. Nevertheless, image-processing techniques allow flatbed scanners to be used in complex tasks, like surface topography characterization of nearly flat objects [14] or contour delineation of three-dimensional objects [15,16]. These devices have several advantages over alternative imaging methods (microscopy, photography …), like a broader field of view or an outstanding relationship between resolution and cost, which make them suitable for a wide variety of enforcements. Examples of the use of flatbed scanners can be found in the field of biomedical imaging [17], materials science [18], astronomy [19] or agriculture [15]. Also in the field of AM, recent works had explored the possibilities of characterizing power defects in power bed fusion processes analyzing digital images captured with a flatbed scanner [20]. In the aforementioned research, attention was paid to the presence of unevenness on the powder surface revealed by out-of-focus regions in the acquired image. This work strengthens the hypothesis that this type of on-machine verification could provide the basis to develop closed-loop manufacturing strategies that minimize the presence of defects on AM parts.

Nevertheless, it is also known that flatbed scanners introduce deformation errors in the digital images that could reduce their suitability as two-dimensional verification devices. This disadvantage has been analyzed by several researchers. Some of these works are focused on the assessment of geometry resolution, defined as the capability of reproducing fine details from the scanned object in the digital image [21]. Although this aspect would be especially relevant if verification of very small features is required, in a first stage of analysis the main efforts should be focused on the reliability of macro-feature digitizing and error correction [22]. Deformation in scanned images has been reported to be caused by mechanical errors in the device [23]. High-precision measuring scale rulers could be used for characterization of defects like non-linearity and deflection of the guide in the vertical plane, non-linearity of the guide in the horizontal plane, eccentricity of the driven guide-roller, bushing skews and positioning error. Moreover, this research also accounts for the additional effect of hindrance sources: scratches, nap, dust particles and particles generated during the wearing out of the scanner parts. Although the overall effect is that of a complex distortion pattern, the authors proposed the simultaneous scanning of the object to be digitized and two standard scales located along the edges in both X and Y directions. Deformation of the digital image of these scales would later be used to compensate the whole image according to a global distortion criterion. Other works [24] revealed that errors along both axes could be non-linear, with a slight s-shaped distortion along the direction of the sensor and a periodic sinusoidal-like distortion in the direction of the scanning movement. In the aforementioned work, the authors proposed the use of a calibration table, composed by a series of calibrated sub-zones where the error curve has been approximated following a linear behavior. An uncertainty analysis according to the Guide to the Expression of Uncertainty in Measurement (GUM) is also provided for the calibrated scanner. Differences regarding measuring direction has also been reported [25]. In this work, the variation in the measured dimensions of a glass scale with photo-etched graduations, calculated from flatbed scanned digital images, has been reported to be related to scale orientation within the scanning area. A 0.5% gradual increase in the measured distance for 1 mm spaced marks between both ends of the 100 glass is registered when the scale is placed parallel to the scan head. Conversely, a 1% periodic variation of the same parameter is registered every 20 mm when the glass scale is placed orthogonal to the scan head. The consequences of image deformation cannot be considered negligible for verification purposes, since other works also reported the existence of periodic errors that could reach 50 µm along the scanning movement direction [26], whereas less relevant errors showing no periodic behavior would be found in the direction parallel to the scan head.

Since image deformation issues had been proved to be inherent to flatbed scanning, a research effort has been paid to deformation modelling and sensor adjustment. De Vicente [27] proposed a model that allows a flatbed scanner to be used as a bi-dimensional coordinate measurement machine. They divided their procedure in two stages: in the initial one they performed an adjustment based on self-calibration [28] of the measuring system where the geometrical errors of the equipment are eliminated; in the final stage a calibration procedure is performed, so that a calibration parameter C_k_ is calculated. This method has been used to measure dimensional features of a flat part with good results. A variation of this method has also been used to compensate flat 3D printed part contours in order to improve the quality of parts manufactured thereafter [29]. Although this method contemplates linear deformations, other authors provided calibration models based on higher order polynomials [30].

A literature review shows that, although commercial flatbed scanners could be a suitable option for two-dimensional verification, they provide digitized images with significant distortion issues. Consequently, distortion would reduce the accuracy of measures taken after processing the images. Reported errors could be minimized by means of scanner adjustment models, which could take the accuracy of measurements towards industrial-degree standards. This approach has been previously used to deal with dimensional errors of 3D parts, but it could be easily adapted to verify two-dimensional geometries. Accordingly, the possibilities of achieving an accurate contour verification of individual two-dimensional layers should be tested in order to lay the groundwork for the future development of closed-loop correction strategies.

Accordingly, the present work explores the suitability of flatbed scanners for two-dimensional verification of AM layer contours. Firstly, scanning distortion and abnormalities concerning commercial contact imaging sensor (CIS)-type flatbed scanners are evaluated and discussed. Then, two different scanner adjustment models are considered and tested by means of a distortion target: one based on compensation of global image deformation and the other based on compensation of local distortions. Finally, the performance of both methods regarding actual AM contours is evaluated by means of the geometrical characterization of circular fused-filament fabricated profiles. For this purpose, results of conventional CMM digitizing of such contours have been used as reference. Scanning operations of AM profiles reflected in this work had been performed off-line.

## 2. Distortion Effects in Commercial Flatbed Scanners

### 2.1. Materials and Equipment

**Figure 1:**
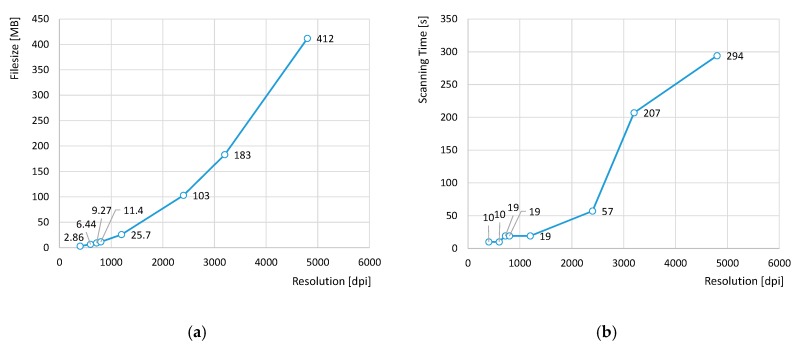
Influence of resolution on file size and scanning time for a 110 mm × 110 mm area: (**a**) Bitmap (BMP) file sizes vs. resolution; (**b**) scanning time vs. resolution.

Distortion issues affecting digital images obtained with a Perfection V39 EPSON flatbed scanner have been analyzed. This model is a low-cost device, with a working area of 216 mm × 297 mm and optical scanning resolution up to 4800 dots-per-inch (dpi) along both axis: sensor axis (parallel to the linear array of photo-detectors) and scanning axis (along the sensor’s movement direction and perpendicular to sensor axis). The theoretical dimensions of the area to be scanned and the desired resolution affect both the size of the resultant image file and the time required for scanning. This effect could be better understood through the graphs in Figure 1, where comparisons between 8-bit greyscale file sizes and scanning times of a 110 mm × 110 mm area at different resolutions are provided.

As can be observed in Figure 1b, scanning time abruptly increases for resolutions over 2400 dpi, lasting 5 min approximately for 4800 dpi resolution scan. A similar trend can be observed for correspondent files size. Nevertheless, whereas high scanning time could be assumed within the scope of this research, files size of several hundred MB turned out to be a major problem for subsequent image processing. Some researchers avoid this problem by reducing image resolution and/or narrowing scanning area [27,31]. In the present work, since considering the whole range of available resolutions was among the initial research purposes, a restriction of maximum allowable scanning area was imposed. Consequently, image size was limited to a 3′′ × 3′′ square area (approximately 76.2 mm × 76.2 mm), which means that maximum scanning time would not exceed 2.5 min and maximum file size should stand under 200 MB.

Image distortion could be evaluated using calibrated grid distortion targets [22,29]. These targets usually consist of equally spaced dots grid, although some target designs could consist of concentric squares or even crossed lines. A diffuse reflectance grid distortion target model 62-952 manufactured by Edmund Optics has been selected for this work. This model provides 40,401 dots arranged in 201 rows and 201 columns that cover a 50 mm × 50 mm area. Thus, the distance between contiguous dots (either in rows or columns) is within 0.25 mm ± 0.001 mm. Each dot has a diameter of 0.125 mm with a tolerance of ±0.003 mm, and has been built with low reflective chrome deposited upon a 3′′ × 3′′ rectangular float glass substrate. This surface diffusely reflects the light back, so no glare should appear on the scanned image.

### 2.2. Target Scanning and Image Processing

Image distortion can be characterized by means of the analysis of differences between dot-to-dot measured distances (as calculated from the scanned target) and their correspondent “true” values (as certified by the target supplier). Nevertheless, calculation of dot-to-dot distances is not a direct task, but requires four consecutive steps: target scanning, image binarization, calculation of centroids and calculation of distances.

Firstly, the distortion target should be placed upside-down on the scanner, so that it lies in contact with the reference sides of the scanner bed. Notice that this does not imply that dot rows and columns would be perfectly aligned with scanner axes P and Q. In fact, it has to be assumed that the target will be slightly tilted. This is not relevant for the adjustment procedures, since they calculate distances between points (which are actually invariants as they do not depend on target orientation), and not their projections with regard to the scanning axes (which depend on target tilting).

The desired resolution should be adjusted before each scanning operation using the software provided by the manufacturer. In this work, all scans are 8-bit greyscale bitmaps (.bmp).

**Figure 2:**
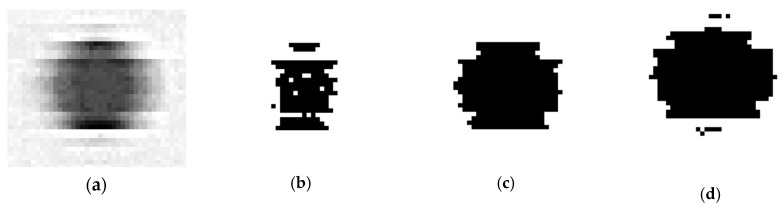
Binary threshold: (**a**) original 256 greyscale levels scan of a dot; (**b**) 2-level image (threshold level = 80); (**c**) 2-level image (threshold level = 165); (**d**) 2-level image (threshold level = 213).

Once scanning has been completed, the images must be processed. In order to do this an application has been developed on C# that employs the image-processing library Emgu CV [32]. First, the 256 levels of grey, ranging from 0 (pure black) to 255 (pure white), of the 8-bit image should be transformed into a two-level black/white image. This process is known as binary thresholding, and involves categorizing greyscale pixels into two classes: black (values below a given threshold level) or white (values over mentioned threshold level). It should be noted that the value of the threshold level determines which pixels would be transformed into pure black or pure white. Figure 2 provides an example of how an 8-bit individual dot image from 4800 dpi scan is transformed into different binary images depending on the selected threshold level.

Although there are different methods available for calculating the threshold level, Otsu´s method [33] has been adopted in the present research. Otsu calculates the optimal grey level threshold by minimizing the intra-class original greyscale values variance. This method is widely considered as the most appropriate option when image histograms show bimodal distributions. Hence, threshold level does not adopt a single fixed value, but should be calculated according to every variation of scanning conditions (e.g., resolution).

**Figure 3:**
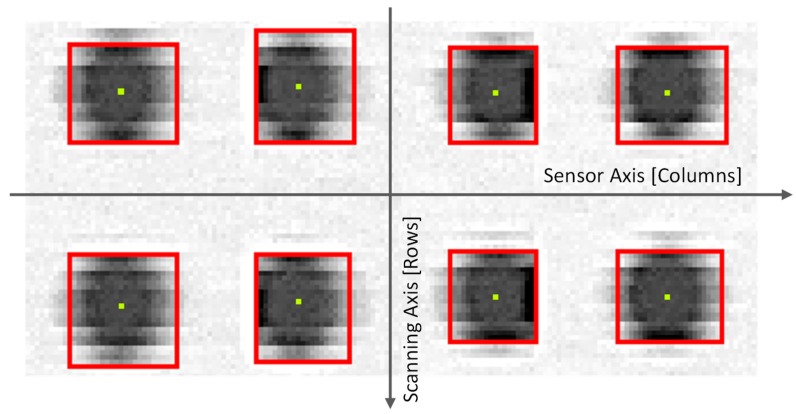
Results from connected-components labelling: original greyscale image, calculated centroid (yellow points) and boundary frames (red lines).

Once binarization is completed and the image transformed into a collection of black spots on a white background, the next step implies identifying accurately isolated dots. This is necessary because the binarization of noisy images could lead the algorithm to split a particular dot into several nearby spots. The connected-component labelling algorithm included on Emgu CV [32] has been used here to evaluate if several spots should be considered part of the same dot and, consequently, if they should be labelled as one unique entity. The algorithm directly provides several descriptive statistics related to each dot. This information includes the location of its centroid with respect to the coordinate system expressed in pixels. Figure 3 contains an image of the original 8-bit scanned image of eight dots (arranged in a two row/four column array) with the superimposed results of binarization and connected-component labelling: red frames surrounding the dots and yellow points at the correspondent centroids.

Accordingly, the connected-component labelling generates a collection of isolated dots that are characterized by the location of their respective centroids. This collection is organized according to a regular grid, so that each single dot is also labelled according to its correspondent row and column. This procedure must be performed before distances between dots can be calculated. An incremental model, which assigns the value of row and column for a given dot with respect to the previous one has been used.

Once the assignment of the collection of dots to a regular grid has been completed, absolute distances between each single centroid and the first one in the corresponding row or column, are calculated independently along rows and columns. This procedure provides two independent arrays. The first array contains the absolute distance $dC_{ij}$ measured between a given dot $D_{ij}$ in a given column (j) with respect to the reference one in the same column $D_{0j}$, by means of the scanner coordinates in axes P and Q. Therefore, the pixel coordinates of $D_{ij}$ are ($p_{ij},q_{ij}$) and the coordinates of $D_{0j}$ are ($p_{0j},q_{oj}$) (Equation (1)). It should be noted that a scale factor based on the selected resolution ($R_{S}$) has been applied to obtain distances in mm from pixel coordinates. The second one contains the absolute distance $dR_{ij}$ measured between a particular dot $D_{ij}\;\left(p_{ij},q_{ij}\right)$ in a given row (i) with respect to the reference one $D_{i0}\;$($p_{0j},q_{oj}$) (Equation (2)). Rows and columns have been labelled from 0 to 200.

(1) $$dC_{ij}\;=\;\frac{25.4}{R_{S}}\;\cdot \;\sqrt{{\left(p_{ij}\;-\;p_{0j}\right)}^{2}\;+\;{\left(q_{ij}\;-\;q_{0j}\right)}^{2}}$$

(2) $$dR_{ij}\;=\;\frac{25.4}{R_{S}}\;\cdot \;\sqrt{{\left(p_{ij}\;-\;p_{i0}\right)}^{2}\;+\;{\left(q_{ij}\;-\;q_{i0}\right)}^{2}}$$

In order to clarify distortion effects, measured distances have been substituted by deviations. This way, the deviation value $\Delta C_{ij}$ for the absolute distance between a particular dot ($D_{ij}$) in a given column (j) with respect to the reference one in the same column ($D_{0j}$) would be calculated as the difference between the distance measured on the image ($dC_{ij}$) and its correspondent theoretical distance ($DC_{ij}$) (Equation (3)). The same procedure has been used in (Equation (4)) for deviations along rows $\Delta R_{ij}$.

(3) $$\Delta C_{ij}\;=\;dC_{ij}\;-\;DC_{ij}$$

(4) $$\Delta R_{ij}\;=\;dR_{ij}\;-\;DR_{ij}$$

### 2.3. Characterization of Image Distortion

Image distortion analysis has been split into two: distortion effects affecting the image along sensor axis direction (evaluated by means of the distance between points in the same row) and distortion effects affecting the image along the scanning axis direction (evaluated by means of the distance between points in the same column).

**Figure 4:**
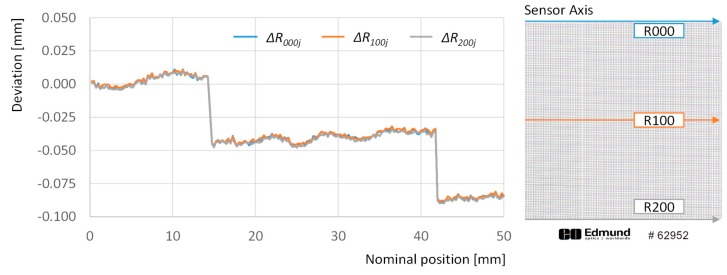
Deviations with respect to the theoretical 0 position calculated for points along rows 000 (blue line), 100 (orange line), and 200 (grey line).

First the deviations observed along dots in the same row are analyzed. The graph in Figure 4 illustrates the deviations for points in rows 000, 100 and 200 across the whole set of columns that cover 50 mm along the sensor axis.

Two abrupt changes in the overall tendency stand out from the less-pronounced fluctuation effects that can also be observed in this graph. Sudden drops of nearly −50 µm have been recorded between the 57th column deviation and the 58th and 59th columns’ deviation. An equivalent phenomenon has been recorded between the 167th column deviation and the 168th one. The accumulative effect drifts the absolute deviation of the last point in each row to a −84 µm approximate value. This implies that the digitized image is contracted with respect to the physical target, but also that this contraction is not regular along the whole length, but the result of a combination of small fluctuations and abrupt changes. Since this effect has not previously been reported in available literature, and this was not an expected phenomenon, a more detailed examination of the image was carried out in order to elaborate a feasible explanation.

**Figure 5:**
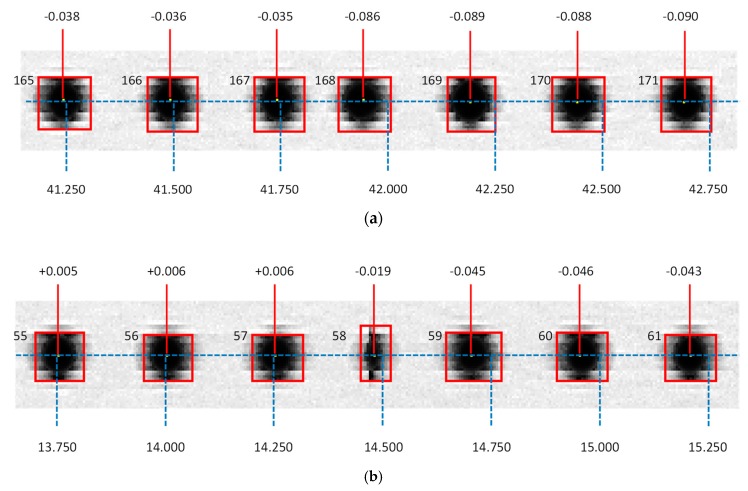
Examples of image plication in the contact imaging sensor (CIS)-type scanner used in present work. Blue dashed lines represent the expected position of each centroid, according to target characteristics, whereas red continuous lines represent the actual position of centroids: (**a**) space inter-dots affected by plication; (**b**) dot affected by plication.

Figure 5 contains an augmented view of points from the first and second rows in the window between the 165th column and the 171th column (Figure 5a) and that between the 55th and 61th columns (Figure 5b). Dots of that figure are presented plainly in the original 8-bit greyscale, as they were actually digitized, and show superimposed their correspondent rectangles, as they are provided by the connected-components labelling algorithm. The actual measured positions are represented by continuous red lines, whereas the expected positions are represented by dashed blue lines.

An abnormal point shape is clearly identifiable regarding points in column 58th (Figure 5b), which results in a discontinuity in the overall trend of deviation values. The effect is similar to that observed after surgical plication, where folding results in shortening a tissue while keeping an apparent continuity. Following this similitude, this effect shall be referred to as image plication from now on. The most probable cause for scan plication seems to be the particular architecture of the CIS-type scanner used in this research. There are two possible arrangements for CIS sensors: in-line structure and staggered structure [34]. The former disposes independent sensors lined-up following a straight line, which implies that, due to the fact that the chip is larger than the outer border of the photo-detectors, an unavoidable physical gap should appear between the last pixel of one sensor and the first of the next one. This circumstance is ignored during the processing of the image file, so that pixels are arranged consecutively, even when they were captured by adjacent sensors with an intermediate gap. This source of error can be presumably avoided using staggered sensor structures that allow an overlapping of the image (overlapped area being captured by two staggered sensors at a time) that is processed in order to avoid gaps.

Two situations could be distinguished regarding plications caused by gaps between sensors: if the plication lays in the intermediate space between dots (like the case of the one located between columns 167th and 168th), its effect will be an evident drop in the expected value for the distance to the origin of the dot in the 168th column with respect to the equivalent value calculated for the dot in the 167th column (Figure 5a). The same will happen to dots in the 169th column and above. On the other hand, if the plication lies directly upon a dot (like the case of the one on the 58th column) the drop will affect completely to the distance calculation of the dot in the 59th column, but also will affect partially to distance calculation of the dot in the 58th column (Figure 5b).

**Figure 6:**
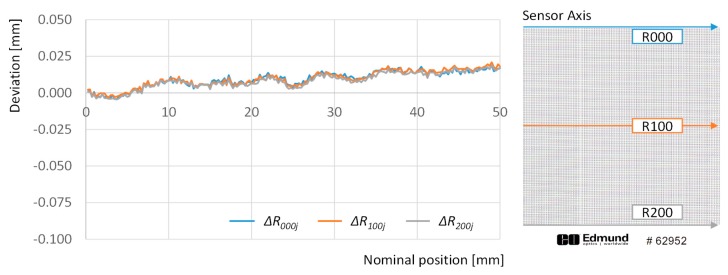
Deviations with respect to the theoretical 0 position calculated for points along rows 000 (blue line), 100 (orange line), and 200 (grey line) after removing gap effect.

Nevertheless, these gaps can be found at the same location in different rows and, having a physical source that is not subjected to variations, their effect could be measured easily and compensated for during the adjustment procedure. To illustrate this, Figure 6 provides a different version of absolute deviations in rows 001, 100 and 200, where abnormal changes related to gaps between sensors have been removed, by substituting dots deviation corresponding to columns 58th, 59th and 168th by their respective expected distances, taking into account the overall tendency.

Once the effect of gaps has been removed, the image appears to be enlarged with respect to the target along the sensor axis in an approximate linear ratio of 0.4 µm per millimeter, which means that a 50 mm object would appear in the image as approximately 18 µm wider. Nevertheless, the graph also shows that this effect is not completely linear: e.g., a linear regression of the deviations for row 200 provides a R^2^ value of 0.793. Additionally, when deviations along the sensor axis direction are compared between different rows, a mere 1 µm average difference is obtained. This means that deformation effects along sensor axis are nearly constant for different positions along the scanning axis.

**Figure 7:**
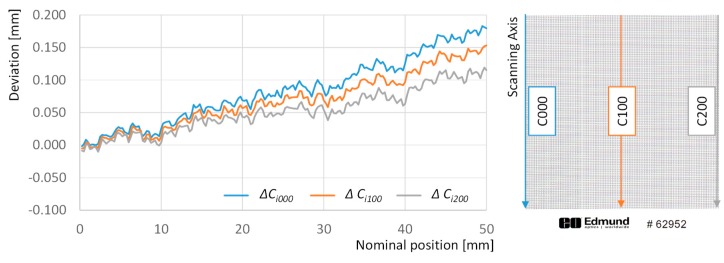
Absolute deviations with respect to the theoretical position of points along columns 000 (blue line), 100 (orange line), and 200 (grey line).

On the other hand, deviations within columns have been also analyzed. The graph in Figure 7 includes such deviations for columns 001, 100 and 200 across the whole set of rows that cover 50 mm in the scanning direction (orthogonal to the direction of the sensor). The first conclusion is that the image of the target is enlarged along the scanning axis with respect to the target itself, and this elongation is progressive and could be considered quasi-linear, even when a combination of slight fluctuations at different frequencies can also be observed. The overall effect is that the absolute deviation of the last point in the first column reaches a +179 µm approximate value, almost doubling the negative distortion along the sensor axis. Another important issue raises from the appreciable differences regarding deviation drift between columns. Image enlargement is greater along the first column than it is along the last one, while the intermediate column shows an intermediate value for the drift. Consequently, the distance between the first and the last points in the first column is 179 µm greater than the theoretical value, whereas this value drops to 153 µm in the case of the 100th column and to 115 µm for the 200th column. This tendency is almost linear and resembles the shape of an open foldable fan.

To evaluate if the described distortion effects are repeatable or not, a specific test has been carried out. Firstly, ten consecutive scans of the dot target have been executed. Those scans have been performed without removing the target from the scanner glass or modifying its position. Then, distances between each point and the reference one have been calculated individually for rows and columns. After that, average values and standard deviations for each single distance were calculated. Results show that the arithmetic mean of the standard deviations calculated for distances along the sensor axis was 1.7 µm, whereas the correspondent standard deviation is 0.9 µm. On the other hand, the arithmetic mean of the standard deviation value in the case of distances along the scanning axis was 1.2 µm, whereas the correspondent standard deviation is 0.7 µm.

This means that the deformation described in previous paragraphs is consistent and the related effects could be considered systematic. A similar result was obtained when the test was repeated removing the target after each scan. This result implies that distortion description could be decoupled between effects along the sensor axis and the scanning axis.

**Figure 8:**
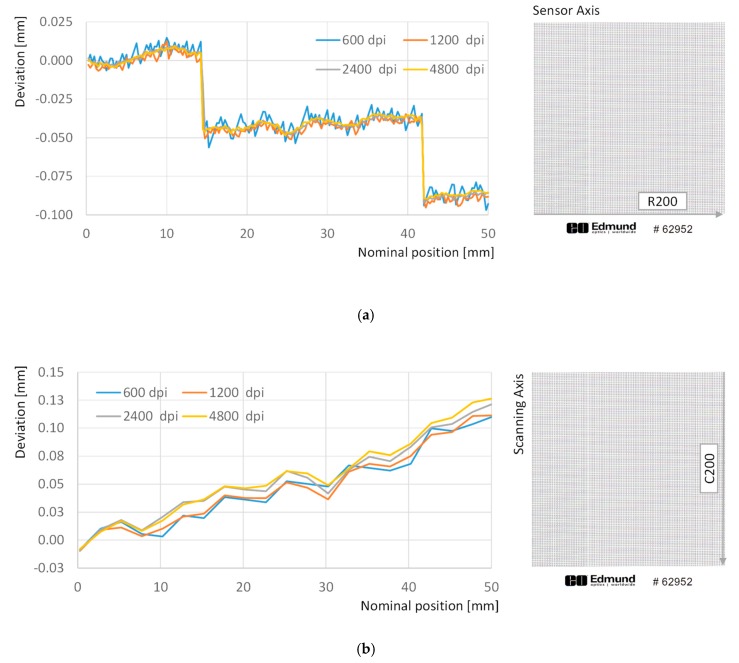
Effect of resolution on deviation values: (**a**) deviations with respect to the theoretical position of dots along row 200; (**b**) deviations with respect to the theoretical position of dots along column 200.

Finally, the influence of scanning resolution upon image distortion has also been analyzed by means of consecutive scans of the test target under 600 dpi, 1200 dpi, 2400 dpi and 4800 dpi resolutions. Results indicate that scanning resolution has different effects upon image distortion between sensor axis and scanning axis. In sensor axis, slight differences can be appreciated, since the overall trend is similar with independence of the resolution, and local differences are related to how the shape of each point is processed during the binarization and connected components steps. In fact, two sources of discrepancy take place simultaneously: differences in dot shape will cause differences on centroid location and coarser resolution will increase the uncertainty of location assessment. Figure 8a shows how resolutions of 4800 dpi, 2400 dpi and 1200 dpi provide quite similar values for deviations along row 200, whereas slight differences can be observed regarding 600 dpi resolution, probably related to how graphical information extracted from dot target is processed. The average difference between deviations for a given column (e.g., number 200) does not exceed 5 µm in the worst case (differences between 600 dpi and 1200 dpi) and are reduced to just 1 µm in the best case (differences between 4800 dpi and 2400 dpi). These differences are consistent along the rows, with standard deviations ranging below 3 µm.

On the other side, results are not so consistent along the scanning direction, since average differences of 9 µm with a standard deviation of 6 µm are found between the 4800 dpi and the 600 dpi resolutions (Figure 8b). Even more, a trend has been observed indicating that discrepancies between resolutions increased with distance to the origin along the columns. In fact, the difference of position for the farthest point (row 200, column 200) reaches 16 µm when 4800 dpi and the 600 dpi resolutions are compared. This effect seems to be derived from a certain discrepancy related to the scanning speed, so that slower scanning speeds (higher resolutions) tend to reflect a higher enlargement of the image in the scanning direction than that observed for faster scanning speeds (lower resolutions).

As a result, it can be concluded that deformation adjustment must be performed individually for each scanning resolution, so that a particular adjustment should be used to compensate image deformation obtained under different resolutions.

## 3. Sensor Adjustment

A process of adjustment and calibration must be performed before the scanner would be used as a contour digitizing device for dimensional and/or geometrical verification of AM parts. In this work, two alternative procedures have been considered.

**Figure 9:**
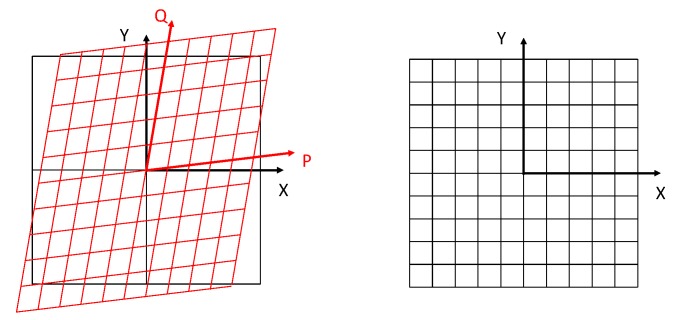
Reference system distortion according to De Vicente and Majarena.

The first method is based on the one proposed by De Vicente [27], as it was thereafter implemented by Majarena [29]. This method assumes that the coordinate system of the flatbed scanner (PQ, see Figure 9) is not a Cartesian one (XY), but in fact formed by a tilted, non-orthogonal axis with dissimilar scales (due to slight differences in pixels size).

Therefore, De Vicente proposed that the geometrical error could be suppressed by measuring a linear-array dot artefact located at multiple positions and using the invariance principle of the measurand (distances between dots are dimensionally stable with independence of the location or orientation of the artefact) to define a function that transforms non-Cartesian coordinates (p,q) into Cartesian ones (x,y). This function can be defined as in Equation (5).

(5) $$\left[\begin{array}{c} x\\ y \end{array}\right]\;=\;\left[\begin{array}{c} p\\ q \end{array}\right]\;+\;\left[\begin{array}{cc} A & \frac{1}{2}\Theta \\ \frac{1}{2}\Theta & -A \end{array}\right]\cdot \left[\begin{array}{c} p\\ q \end{array}\right]$$

where A is a coefficient that represents the relative differences between pixel dimensions, according to the P and Q axes, and Θ represents the lack of perpendicularity between these two axes. Solving by an ordinary least squares problem this equation for all the measurements done on the artefact, estimators for both parameters “A” and “Θ” are calculated. Then, using a calibrated line-scale standard, a calibration parameter $C_{k}$ is also calculated (Equation (6)), providing the desired metrological traceability.

(6) $$\left[\begin{array}{c} x\\ y \end{array}\right]\;=\;\left[\begin{array}{c} p\\ q \end{array}\right]\;+\;\left[\begin{array}{cc} C_{k}\;+\;A & \frac{1}{2}\Theta \\ \frac{1}{2}\Theta & C_{k}\;-\;A \end{array}\right]\cdot \left[\begin{array}{c} p\\ q \end{array}\right]$$

In the formulation followed by Majarena, both the adjustment and calibration steps have been unified using a single grid-target calibrated standard, which allows for using known distances between points in different directions (including diagonals) from a single scanning operation. In such a way, the parameter A includes both the effect of dissimilarities between pixel dimensions (geometrical error) and that of the scale (dimensional error). Since this method aims at adjusting the overall geometrical error of the scanned image, it will be referred to as the global distortion adjustment (GDA).

The second method explores an alternative approach: instead of using a global parametric model in which optimal values for each parameter are obtained by adjusting experimental data, those data are used to build a local interpolative model. This model would no longer be based on the relationships between both systems taken as a whole, but to the individual correspondence between the coordinates of the same point expressed with respect to both systems. Therefore, the objective is to define a collection of discrete functions capable of accurately computing the distortion of the image without neglecting local effects.

Instead of using a single model for the overall distortion, the problem has been split into two independent ones: distortion along X and distortion along Y. Therefore, two independent maps of distortion were built. The first one reflects the real position of points with respect to the X direction, as a function of the coordinates p and q (expressed with respect to the flatbed scanner reference system). In a similar way, the second one reflects the real position of those points with respect to the Y direction. It has to be considered here that the positions of each dot centroid, referred to the flatbed system, would not be arranged in a regular grid because of the distortion of the image, even when the physical target could be considered as nearly-regular grid. Consequently, the most logical approach here should be to consider a model that allows for interpolation of scattered data at the local level.

**Figure 10:**
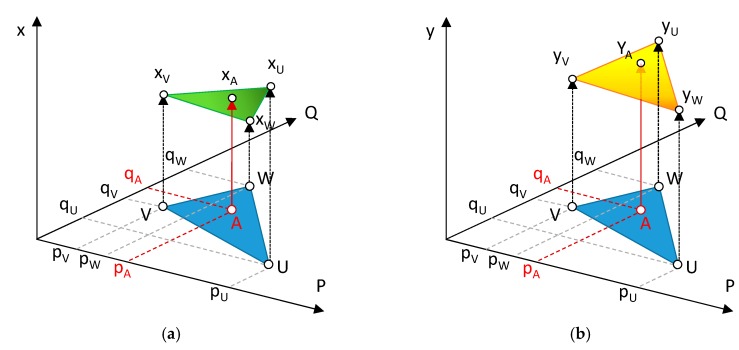
Decoupled interpolation model: (**a**) *x* coordinates; (**b**) *y* coordinates.

In the present work, a linear interpolation triangle-based model has been used. Firstly, a Delaunay triangulation is performed. This process generates a collection of non-intersected triangles that cover the whole interpolation area. In order to obtain the position of a point A ($p_{A}$,$q_{A}$) from a scanned image expressed along the X coordinate of a XY Cartesian system, the algorithm firstly identifies the triangle where the point is located, defined by three vertex (U, V, W), in the map of distortion for the X direction (Figure 10a). Then using the corresponding x coordinates for these three points obtained from the interpolation model ($x_{U}$,$x_{V}$,$x_{W}$), the system formed by Equations (7)–(9) is obtained:

(7) $$x_{U}\;=\;a_{0}\;+\;a_{1}\;\cdot \;p_{U}\;+\;a_{2}\;\cdot \;q_{U}$$

(8) $$x_{V}\;=\;a_{0}\;+\;a_{1}\;\cdot \;p_{V}\;+\;a_{2}\;\cdot \;q_{V}$$

(9) $$x_{W}\;=\;a_{0}\;+\;a_{1}\;\cdot \;p_{W}\;+\;a_{2}\;\cdot \;q_{W}$$

Thus, coefficients $a_{0}$, $a_{1}$ and $a_{2}$ are calculated and Equation (10) is obtained.

(10) $$x\;=\;a_{0}\;+\;a_{1}\;\cdot \;p\;+\;a_{2}\;\cdot \;q$$

This plane represents the expected values for the X coordinate of every point located inside the triangular region defined by points U, V and W, considering that deformation can be assumed as approximately linear within its limits (Figure 10a).

A similar system would thereafter be constructed for the Y coordinate (Figure 10b). Accordingly, the position of every point A with respect to the Cartesian system ($x_{A}$,$y_{A}$) calculated with Equations (11) and (12).

(11) $$x_{A}\;=\;a_{0}\;+\;a_{1}\;\cdot \;p_{A}\;+\;a_{2}\;\cdot \;q_{A}$$

(12) $$y_{A}\;=\;b_{0}\;+\;b_{1}\;\cdot \;p_{A}\;+\;b_{2}\;\cdot \;q_{A}$$

Similar functions would be constructed in order to cover the whole area defined by the dot target. The result is a series of functions that show continuity along the edges of the triangular areas, but do not contemplate second or higher order adjustments. Since this method aims at adjusting local geometric errors upon the scan, it will be referred to as the local distortion adjustment (LDA).

**Figure 11:**
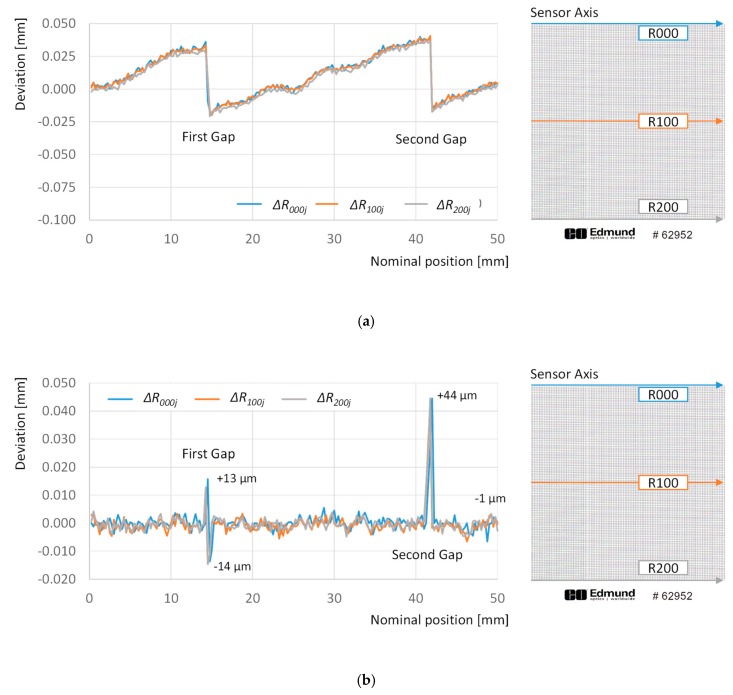
Deviations with respect to the theoretical 0 position calculated for points along rows 000 (blue line), 100 (orange line), and 200 (grey line): (**a**) after adjustment with global distortion adjustment (GDA); (**b**) after adjustment with local distortion adjustment (LDA).

Both GDA and LDA methods were implemented, and the parameters ($a_{0}$, $a_{1}$ and $a_{2}$; $b_{0}$, $b_{1}$ and $b_{2}$) of each necessary function calculated by means of a reduced set of points and columns. In order to reduce computational cost, target resolution was artificially reduced to 1 mm × 1 mm by selecting only one of each four rows or columns. This decision reduced from 40,401 to 2601 the number of points used for adjustment. Once the alternative adjustment models were computed, they were applied to a new scanning of the grid standard. Although both alternatives provided a reduction in geometric distortion, there were clear differences regarding how this goal was achieved. Firstly, Figure 11 provides two graphs reflecting how absolute deviations with respect to the theoretical position of points along different rows have been modified by each adjustment model.

The GDA compensates the overall drift observed in Figure 6, reducing the average deviation value from to −35 µm to 10 µm, and the accumulated drift at the end of the rows from (approximately) −84 µm to 4 µm. Nevertheless, these values do not properly reflect the behavior observed in Figure 11a, since the adjusted distances present an overall upward trend that suffers two clear downward corrections at the sensor gaps. On the other side, LDA reduces the average deviation to −0.06 µm and the accumulated drift at the end of the rows to −1 µm. Unlike GDA, this correction is almost uniform along the rows, but it is not capable of properly compensate the location of points in the influence area of the gap effect. Accordingly, a maximum deviation of +44 µm is observed as a peak in the vicinity of the second gap, and a fluctuation of +13 µm to −14 µm is observed in the vicinity of the first one.

**Figure 12:**
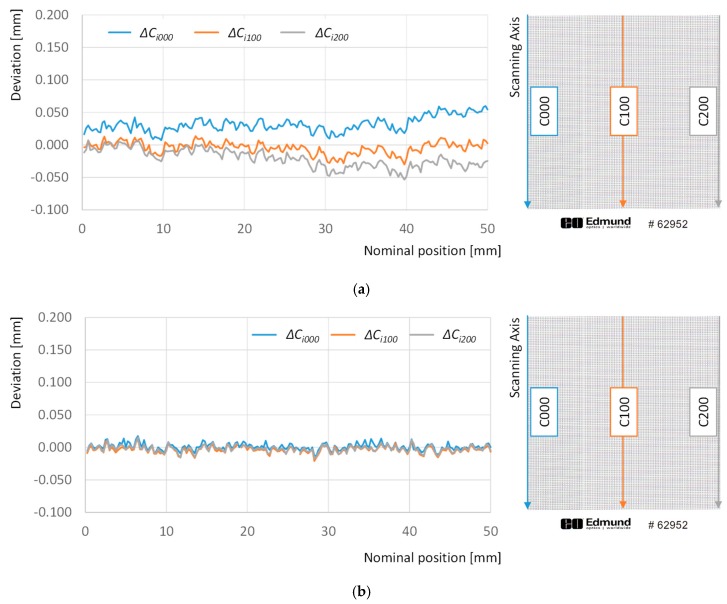
Deviations with respect to the theoretical 0 position calculated for points along columns 000 (blue line), 100 (orange line), and 200 (grey line): (**a**) after adjustment with GDA; (**b**) after adjustment with LDA.

A similar analysis has been performed for absolute deviations with respect to the theoretical position of points along different columns (Figure 12).

Adjustment via GDA almost suppresses the enlargement trend in absolute deviations along columns, as the absolute deviation observed for the last point of the first column is reduced from +179 µm to +54 µm, and this effect could also by observed in the other columns in the graph. Nevertheless, it can be also noticed that the *open foldable fan* effect observed in Figure 7 could not be corrected properly. In fact, deviations of points in the first column show a slight increase of, approximately +25 µm along the 50 mm length. At the same time, the average value for deviations along the 100th column is only +5 µm, showing no significant trend. Finally, deviations of points in column 0 show a slight decrease of, approximately −25 µm along the 50 mm length. On the other side, LDA reduces the average deviation of points in those columns to −1.6 µm, while showing no significant trend, with independence of the analyzed column. Consequently, LDA does not only suppresses the elongation trend, but also the *open foldable fan* effect.

Comparing both models, it seems clear that the LDA provides better results. Although the gap effect has a negative impact on the capacity of GDA to compensate geometric deformation accurately it has been also observed that even in the other direction (where there are no gaps affecting the results), the particular geometric deformations introduced by the scanner characteristics can be also better compensated for by using a local deformation approach. These results are particularly relevant when the goal is to accurately verify the contour of the part and not just the overall dimension. However, this assertion should be checked upon a real case.

## 4. Case Study

**Figure 13:**
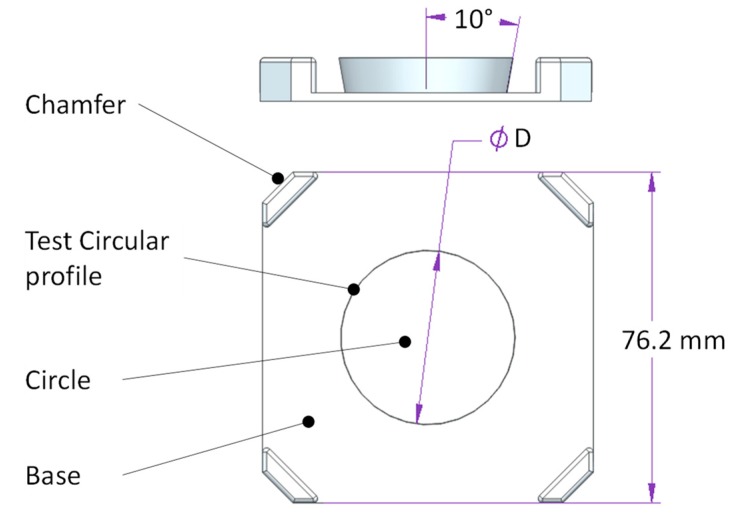
Basic geometry of test specimens.

A series of test specimens have been designed, manufactured and measured in order to properly evaluate the actual differences between GDA and LDA and their relative influence upon the quality of dimensional and geometric verification of a real AM part. A circle has been selected as a test profile whereas the three-dimensional geometry of test specimen is that of the inverted frustum of a right-circular cone, where the upper base (the biggest circular section) is contained in the top layer (the last one to be manufactured). This geometry prevents visualization of underlying layers contours. Additional features have been included in test specimens, as can be observed in Figure 13.

Each test specimen includes a square base of 76.2 mm × 76.2 mm, to match the external dimensions of the target used for error modelling. Each corner is chamfered so that there would be no concerns related to specimen location on the flatbed scanner surface. The inverted frustum that contains the test circular profile has been placed at the geometric center of each specimen. When the part is located upside-down on the scanner glass, the digitized image contains the upper layer of the corresponding circle, surrounded by the top layer of the base. Three specimen sizes were considered in this study, designed as S1, S2 and S3 with diameters (D) of 20 mm, 30 mm and 40 mm respectively.

**Figure 14:**
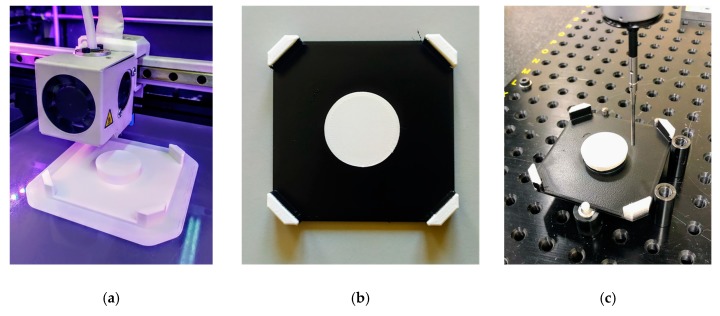
Tests specimens: (**a**) manufacturing; (**b**) final aspect of specimen, once the top layer of the base had been painted in black; (**c**) measuring the geometry in a coordinate measuring machine (CMM) (right).

A BCN3D Sigma fused filament fabrication (FFF) machine has been used for manufacturing of test specimens (Figure 14a), while 2.85 mm thermoplastic filament made of polylactic acid (PLA) was selected as raw material. Layer resolution had been fixed to 0.1 mm, while a 0.4 mm nozzle was used. Once, the specimens were manufactured, the top layer of the base was painted black, to provide an increased contrast with respect to the white PLA of the upper layer (Figure 14b). To obtain an accurate reference of each specimen top layer contour, as they were actually manufactured, all specimens were measured in a DEA Global Image 09-15-08 CMM (Figure 14c).

This machine was calibrated according to EN 10360-2:2001 being the maximum permissible error in length measurement as in Equation (13):

(13) $$MPE_{E}\;=\;2.2\;+\;3\cdot L/1000\;\left(µm\right)\;\left(L\;in\;\mathrm{mm}\right)$$

and the maximum permissible error in probing repeatability as in Equation (14):

(14) $$MPE_{P}\;=\;2.2\;\left(µm\right)$$

In order to properly digitize the contour of the last layer, a cylindrical shank probe (2 mm diameter) was used. Since the end of the probe is semi-spherical, and it was demanding to assure that the probe touched the contour of the top layer with the cylindrical section, a margin of 0.5 mm between the expected location of that contour and the transition between cylindrical and spherical sections of the probe was set in the verification program. A discrete-contact probing strategy was defined so that 360 points (one every 1°) were digitized upon the contour, with the independence of each test specimen size. PC-DMIS^®^ metrology software was, thereafter, used to calculate diameter and center location of each specimen according to a least-squares fitting calculation. With this information and the relative position of each digitized points, radial deviation with respect to the theoretical radius could also be calculated. As mentioned, CMM measurements were used as reference to evaluate results provided by the flatbed scanner under different adjustment methods.

**Figure 15:**
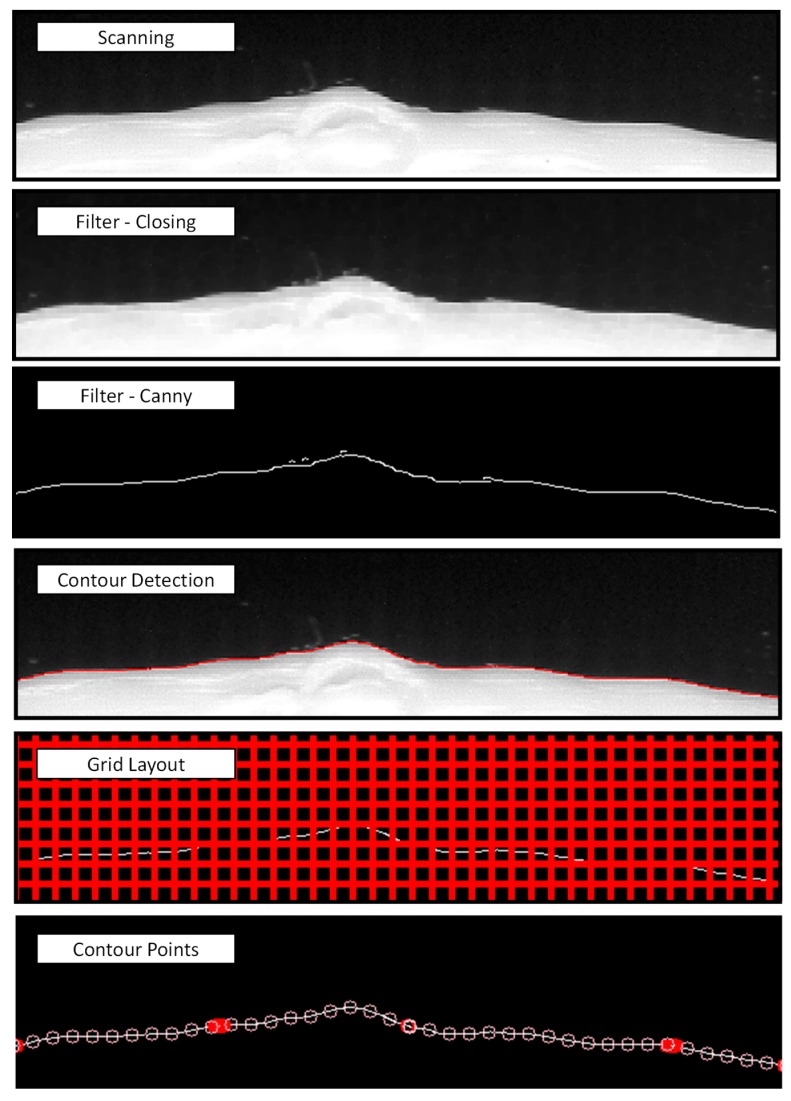
Contour digitizing sequence.

The final step was to digitize test specimens with the flatbed scanner. Accordingly, they were placed upside-down, so that the last manufactured layer lied directly upon the glass, and the border of the specimen was in contact with the lateral edges of the scanner. Thereby, the central geometry of each specimen was located approximately centered with respect to the 50 mm × 50 mm calibrated area. Then, scanning was carried out at 2400 dpi resolution and three greyscale BMP files containing the images of test geometries were obtained. Figure 15 illustrates the contour digitizing sequence, particularized for a small area belonging the upper section of the S3 specimen. After the scanning, a closing-type morphological filter was applied to the resultant greyscale image in order to remove small abnormalities in objects expected to be uniform. These defects could be caused by material behavior during printing, since small quantities of melted plastic could ooze from the nozzle and stick to the contour. This effect is often referred to as stringing and leaves whisker-like structures. In the present work, the closing filter has been found to be useful in order to remove these small isolated clear areas within the dark foreground, allowing for a better detection of part contour. Regarding the parameters of the closing filter, a rectangular Kernel (five-pixel sized) and one single iteration was used. The resultant image was then processed by a canny edge-detection algorithm. Parameters used in canny filtering include size 5 for the kernel of the Gaussian filter, a 150 value for the threshold edge, a 50 value for the threshold link.

Once the application of Canny algorithm has provided a collection of contours, a routine that finds out external contours and suppress small isolated lines was applied. After the contour has been defined, a 15 × 15 pixel grid was overlaid on the image, and intersections between grid and contour were calculated. As a result, a series of contour points were identified and characterized by their coordinates (in pixels) with respect to the scanner origin reference. These points were finally exported to a TXT file.

Once the collection of points and its respective pixel coordinates were obtained, coordinates of every single point were processed under three alternative adjustment procedures. The first one involved using the theoretical dimensions of pixels, which means that no deformation adjustment was applied, but a simple theoretical scale adjustment (TSA). The second one used the GDA based on works by De Vicente and Majarena. The third one applied the LDA proposed in this work as an alternative method, according to the description given in the previous section.

**Figure 16:**
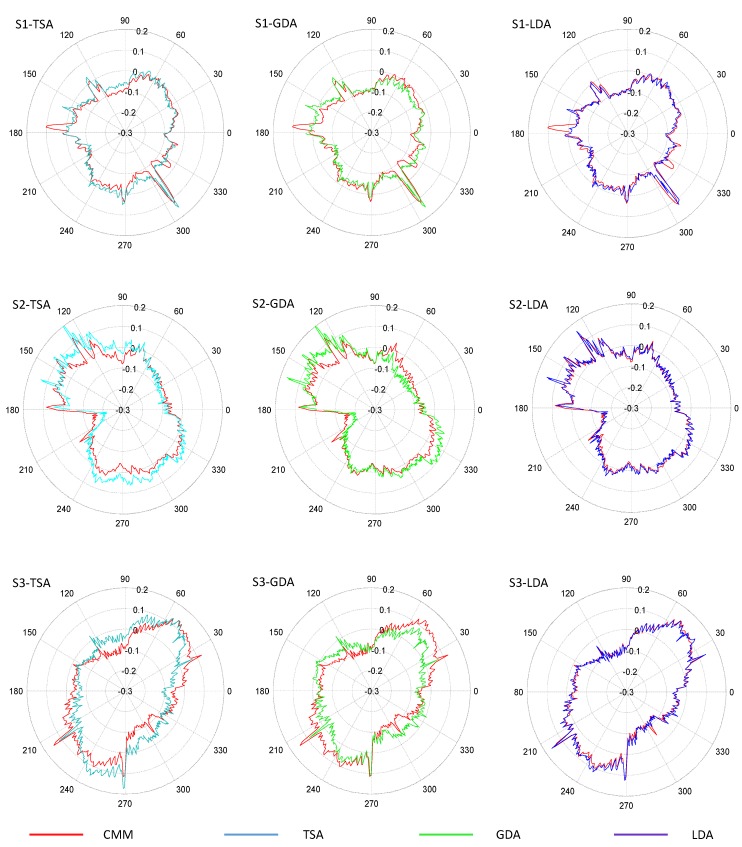
Comparative of radial deviations with respect to the nominal radius for S1, S2 and S3 under different adjustment methods.

To illustrate differences between these three methods, polar graphs showing the radial deviations obtained from the application of the different adjustments, as well as the CMM results taken as reference, are provided in Figure 16 for each test specimen. Colored lines in Figure 16 represent deviation calculated for points digitized with the flatbed scanner after different adjustments: TSA (light blue), GDA (green) and LDA (dark blue), whereas the red lines represent the radial deviation of points captured with CMM with respect to the nominal diameter of the feature.

**Figure 17:**
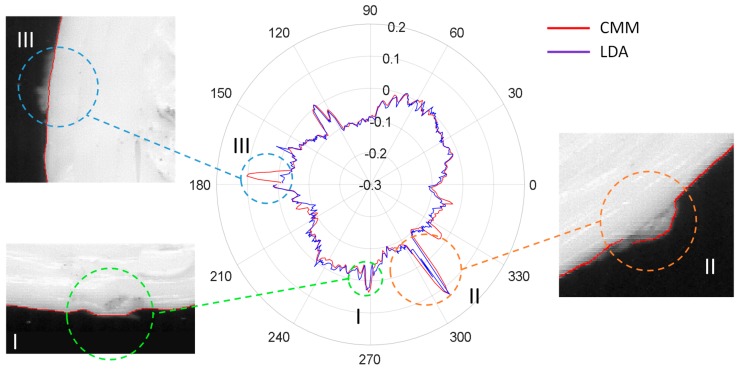
Relevant contour defective features in S1: seam (feature I), small disturbance (feature II) and “ghostly” contour (feature III).

Results revealed that contours processed with LDA are clearly more similar to those digitized by the CMM than contours processed with GDA or TSA. In fact, GDA is not capable of accurately removing deformation effects and thus the green contours fluctuate inside and outside the limits of the red reference contour obtained with the CMM. This means that radial deviation could be either underestimated or overestimated, since GDA does not reflect accurately the actual contour. On the other hand, radial deviations under LDA are quite similar to those used as reference, at least in the cases of S2 and S3. Nevertheless, in the case of S1, there are some abnormalities that should be discussed. Actually, S1 shows located discrepancies between CMM reference profile and adjusted profiles with independency of the considered adjustment. To illustrate this phenomenon, Figure 17 provides several detailed views of the part contour.

The presence of a lateral seam along the vertical walls of FFF parts is a well-known phenomenon, related to the combined dynamics of material flowing from the nozzle and XY movement jerk when starting and ending external contour trajectories. An excess of material is poured out of the nozzle resulting in a small protuberance in each layer. When the start/end position of contours are approximately located at a similar XY for consecutive layers, a seam-like feature (similar to a welding seam) appears. As can be notice in Figure 17, the LDA contour matches the seam shape (feature I) with an accuracy similar to that of the CMM. Moreover, small disturbances in the expected profile that can also be observed in Figure 17 had been also accurately identified in the LDA profile (feature II), being nearly equal in magnitude as their correspondent value from the CMM measurement. 

Nevertheless, there are at least three features that could be identified easily upon CMM data that do not appear in the scanned profiles, regardless of the method used for adjustment. The most relevant of these “ghostly” features has been labelled in Figure 17 as feature III. Since the adjustment has nothing to do with this discrepancy (the mentioned feature can also be observed in Figure 16 for TSA and GDA contours), the source should necessarily be related to the contour tracing stage. A deeper look at Figure 17 reveals that, in fact, a blurred lighter area can be identified on the digitized image at the expected position. Actually, a small protuberance can be noticed upon the 3D part at that position, but it is located slightly under the top contour; that is to say, this abnormality does not belong to the top layer, but to lower layers. Since the CMM touch probe has been programmed to follow the contour at a 1.5 mm below the top layer, this feature reflects an abnormality that is big enough to overpass the projection of top contour and consequently being wrongly included in the profile, as digitized by touch-trigger technology. This fact reinforces the robustness of the flatbed scanner performance, since the final objective is to accurately digitize last layer contour and avoid, as much as possible, noise introduced by features in previous layers. Since the small protuberance in Figure 17 is clearly blurred and its greyscale intensity lower than that of the points of the top profile, further filtering strategies could be developed.

**Figure 18:**
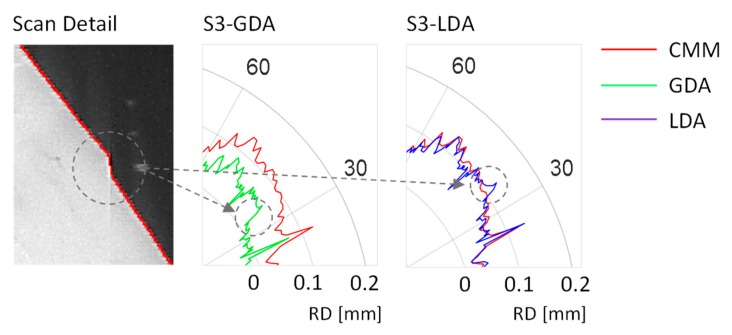
Detail of the plication effect affecting the contour of S3.

Figure 18 provides a partial view of the S3 contour, in the vicinity of one of the gap plications described in Section 2.3. Plication can be clearly observed in the image, and its effect can also be noted in the detailed view of the radial deviation graphs that are also provided in Figure 18. Plication can be noted as an abrupt drop in the contour obtained with GDA, whereas it resembles a peak in the contour provided by LDA. A punctual abnormality is unavoidable under both approaches, but the relevance of the gap is completely different, since LDA (working locally) restrains the gap influence upon adjustment to a small section of the contour whereas, on the other hand, its effect influences the whole compensation under GDA.

In order to compare results, the accuracy of contour tracing can be analyzed by means of the average value of the radial deviations between points measured with the CMM and those measured with the flatbed scanner Accordingly, the circumference has been segmented into 360 angular sectors (1° each), and then the absolute value of the difference between the value of the radius calculated from the scanned images and measured by the CMM has been calculated for each segment. Next, the mean radial deviation RD_M_ is calculated. Results are provided in Table 1.

Results in Table 1 indicate that LDA outperforms GDA when the goal is to accurately describe the contour of the layer. $RD_{M}$ value calculated after GDA is approximately 46% less accurate than that calculated after LDA in the case of S1, whereas the obtained results is 188.7% worse in the case of S2, and 300% worse for S3. Surprisingly, GDA and TSA provide similar values for $RD_{M}$, which means that a global adjustment strategy is not an adequate option for an accurate description of part contour.

Similarly, the consistency between profile fluctuation as measured with the CMM and that obtained with the scanner can be analyzed by means of the standard deviation of the 360 radial deviations previously calculated ($RD_{\sigma }$). Results are provided in Table 2.

$RD_{\sigma }$ results are quite similar for S1, whereas S2 and S3 show better behavior in the case of LDA. This implies that fluctuations of radial deviations with respect to the reference CMM-digitized contour are less severe if a local adjustment strategy is used. These results can be explained by means of the inaccuracy that the gap plication effect introduces in GDA, whereas LDA is far less sensible to the influence of such an abnormality.

## 5. Conclusions

This paper presents an analysis of the use of flatbed scanners for AM layer contour verification. A distortion target has been used to describe how the image provided by the scanner is distorted with respect to the actual shape. It has been observed that distortion effects can be decoupled between sensor axis and scanning axis directions. The overall tendency in both axes is to enlarge images, but gap effects related to CIS in-line sensor architecture actually introduce image plications along the sensor axis, resulting in a global contraction of the image in that direction. It has also been observed that deformation along the sensor axis is similar for different positions of the sensor along the scanning axis, whereas deformation differs along the scanning axis for different sensor axis positions. Additionally, although a certain influence of scanning resolution has been observed, distortion effects are consistent and repeatable when individual points are considered. Different sensor adjustments have been analyzed, and it has been proved that the GDA approach has provided worse results than the LDA approach regarding accurate tracing of contours. Results indicate that local distortions severely affect contour-detection, but this effect could be reasonably avoided by applying a LDA like the one proposed in this paper. As a consequence, the possibility of using flatbed scanners for on-machine measurement of AM layers seems to be feasible, but GDA should not be used for CIS-type flatbed scanner adjustment due to its sensitivity to gap plication effects. In future works, efforts should be conducted in order to develop an on-machine verification procedure able to evaluate each single layer before the next one is deposited and, consequently, providing capabilities for individual layer verification that resemble those of computer tomography. This procedure will also allow for implementing closed-loop strategies that will minimize dimensional and geometrical errors in AM parts. Nevertheless, there are still several concerns that should be addressed. For example, it should be necessary to investigate how distortion adjustment should be extended to the whole scanning area. High-accuracy targets are only available for small areas, so efforts should be conducted to manufacture bigger targets with similar accuracy or to develop a procedure for covering the whole scanning area by means of placing the target at different positions. Nevertheless, this would require developing a procedure to build a complete adjustment from different partial independent adjustments. Finally, the influence of several sources of error (i.e., parameters in contour-detection algorithms or target image processing for dot-to-dot distance calculation) should be analyzed in order to obtain robust results under the expected variety of actual manufacturing conditions.

## Figures and Tables

**Table 1:** Mean radial deviation ($RD_{M}$), calculated from CMM measurements, applying Theoretical Scale Adjustment (TSA), Global Distortion Adjustment (GDA), and Local Distortion Adjustment (LDA.)

$RD_{M}[µm]$	CMM	TSA	GDA	LDA
S1	0	17.6	18.1	12.4
S2	0	29.4	28.0	9.7
S3	0	40.7	36.8	9.2

**Table 2:** Standard deviation of radial deviations ($RD_{\sigma }$).

$RD_{\sigma }\;[µm]$	CMM	TSA	GDA	LDA
S1	0	16.1	14.6	17.3
S2	0	20.3	21.1	10.2
S3	0	23.7	24.7	10
